# Emodin regulates neutrophil phenotypes to prevent hypercoagulation and lung carcinogenesis

**DOI:** 10.1186/s12967-019-1838-y

**Published:** 2019-03-18

**Authors:** Zibo Li, Yukun Lin, Shuhui Zhang, Lin Zhou, Guixi Yan, Yuehua Wang, Mengdi Zhang, Mengqi Wang, Haihong Lin, Qiaozhen Tong, Yongjian Duan, Gangjun Du

**Affiliations:** 1 Institute of Pharmacy, Pharmacy College of Henan University, Jinming District, Kaifeng, 475004 Henan China; 2 School of Pharmacy and Chemical Engineering, Zhengzhou University of Industry Technology, Xinzheng, 451150 Henan China; 3 Hunan University of Chinese Medicine, Changsha, 410208 Hunan China; 4 Department of Oncology, The First Hospital Affiliated to Henan University, Kaifeng, 475001 Henan China

**Keywords:** Emodin, Neutrophil phenotypes, NETs, Hypercoagulation, Carcinogenesis

## Abstract

**Background:**

Hypercoagulation and neutrophilia are described in several cancers, however, whether they are involved in lung carcinogenesis is currently unknown. Emodin is the main bioactive component from Rheum palmatum and has many medicinal values, such as anti-inflammation and anticancer. This study is to investigate the contributions of neutrophils to the effects of emodin on hypercoagulation and carcinogenesis.

**Methods:**

The effects of emodin on neutrophil phenotypes were assessed by cell proliferation, morphological changes, phagocytosis and autophagy in vitro. The anti-coagulation and cancer-preventing actions of emodin were evaluated in the urethane-induced lung carcinogenic model. The expressions of Cit-H3 and PAD4 in lung sections were assessed by immunohistochemistry, CD66b^+^ neutrophils were distinguished by immunofluorescence, and cytokines and ROS were examined with ELISA. The neutrophils-regulating and hypercoagulation-improving efficacies of emodin were confirmed in a Lewis lung cancer allograft model. The related targets and pathways of emodin were predicted by network pharmacology.

**Results:**

In vitro, emodin at the dose of 20 µM had no effect on cell viability in HL-60N1 but increased ROS and decreased autophagy and thus induced apoptosis in HL-60N2 with the morphological changes. In the urethane-induced lung carcinogenic model, before lung carcinogenesis, urethane induced obvious hypercoagulation which was positively correlated with lung N2 neutrophils. There were the aggravated hypercoagulation and lung N2 neutrophils after lung carcinoma lesions. Emodin treatment resulted in the ameliorated hypercoagulation and lung carcinogenesis accompanied by the decreased N2 neutrophils (CD66b^+^) in the alveolar cavity. ELISA showed that there were more IFN-γ, IL-12 and ROS and less IL-6, TNF-α and TGF-β1 in the alveolar cavity in the emodin group than those in the control group. Immunohistochemical analysis showed that emodin treatment decreased Cit-H3 and PAD4 in lung sections. In the Lewis lung cancer allograft model, emodin inhibits tumor growth accompanied by the attenuated coagulation and intratumor N2 neutrophils. Network pharmacology indicated the multi-target roles of emodin in N2 neutrophil activation.

**Conclusions:**

This study suggests a novel function of emodin, whereby it selectively suppresses N2 neutrophils to prevent hypercoagulation and lung carcinogenesis.

## Background

Due to being diagnosed at advanced stages, lung cancer is the leading cause of cancer-related death [1]. In spite of revised and aggressive approaches to therapy, the 5-year survival rate for advanced stage lung cancer has not improved significantly [2]. As is the case for other types of cancer, the normal lung cells spend several years transforming into early precursor lesions and finally into lung cancer, which provides us with several opportunities for preventing or reversing the progression of precancerous lesions [3]. Undoubtedly, the application of chemopreventive agents may decrease the cancer incidence, as well as treatment cost, and contribute to the improvement of patient survival and quality of life [4]. The herb-derived compounds display an unparalleled potential for the proposed development of preventive pharmaceutics [5]. Emodin is a natural anthraquinone present in various Chinese medicinal herbs and is known as the main bioactive component in Rheum palmatum which has been used for over 2000 years in China [6]. Pharmacological research indicated that emodin has a variety of functions, including anti-tumor and anti-inflammation [7, 8]. Previous studies have shown that emodin possesses anti-proliferative function in various cancer cells in vitro and in vivo [9, 10]. A recent study revealed that emodin could also exert tumor preventive efficacy in DMBA-induced oral carcinogenesis [11]. Furthermore, the injury preventive action of emodin was confirmed in several conditions, such as pancreatitis [12], ulcerative colitis [13], intestinal mucosal injury [14] and lung injury [15]. These findings strongly suggest that emodin may protect organ injury against tissue carcinogenesis, however, the different conditions and the involved mechanism need to be further investigated.

Recently, neutrophils, which are the most important effectors of innate immunity, have been shown to have a crucial role in tumor initiation and progression by releasing cytokines, chemokines, ECM remodeling enzymes, and so on [16]. Neutrophils accumulate in many kinds of human and murine tumors and participate in nearly all steps of tumor progression [17, 18]. In clinical practice, the neutrophils denote heterogeneous populations in response to inflammatory stimuli, Fridlender et al. proposed the paradigm of anti-tumoral “N1 neutrophils” versus pro-tumoral “N2 neutrophils” based on neutrophil diversity and plasticity [19]. Most recently, it has been known that neutrophil extracellular traps (NETs) are an important effector mechanism by which neutrophils elaborate response to numerous stimuli [20]. Cancer patients are commonly affected by thrombotic events induced by tumor-related platelet activation [21]. Several studies have suggested an important role of NETs in cancer progression and tumor-associated thrombosis [22–24]. These studies have indicated the complex relationship among neutrophils, hypercoagulation and carcinogenesis. It was reported that spontaneous intestinal tumorigenesis correlated with the accumulation of low density neutrophils with a pro-tumorigenic N2 phenotype, and that hypercoagulation and neutrophilia are frequent incidents in cancer patients [25]. It was also reported that emodin promoted polymorphonuclear neutrophil apoptosis in pancreatitis-associated acute lung injury [26] and ameliorated diesel exhaust particles-induced impairment of vascular and cardiac homeostasis [27]. However, to date, whether hypercoagulation involved in lung carcinogenesis and whether the cancer preventive efficacy of emodin is associated with its antithrombosis are currently unknown. In this study, we explored the relationship between hypercoagulation and carcinogenesis and investigated the contributions of neutrophils to the effects of emodin on hypercoagulation and carcinogenesis.

## Materials and methods

### Materials

Emodin (purity > 98%) was purchased from Xi-an Helin Biological Engineering Co. (Xi-an, Shanxi, China). Urethane, phorbol 12-myristate 13-acetate (PMA), 4′,6-diamidino-2-phenylindole (DAPI) and 2′,7′-dichlorodihydrofluorescein (DCFH-DA) diacetate were purchased from Sigma Chemical Co (St.Louis. MO. USA). Ly6G microbeads, CD66b microbeads and Ly6G monoclonal antibody (Ly6G mAb) were from Miltenyi. Antibodies including anti-CD66b, anti-histone Cit-H3, anti-PAD4, FITC-conjugated anti-mouse CD66b were obtained from BD Pharmingen. Horseradish peroxidase (HRP)-conjugated goat anti-mouse IgG polyclonal antibody, peroxidase substrate DAB (3,3′-diaminobenzidine) were obtained from Nichirei Bioscience (Tokyo, Japan). Annexin V-FITC Apoptosis kit and mouse quantitative ELISA kits (IFN-γ, ROS, IL-12, TNF-α, IL-6 and TGF-β1) were obtained from R&D Systems. Four coagulation kits were obtained from Nanjing jiancheng bioengineering institute. Standard rodent chow was purchased from Henan Provincial Medical Laboratory Animal Center (Zhengzhou, China), License No. SCXK (YU) 2015-0005, Certificate No. 41000100002406.

### Cell culture and assays

The human acute promyelocytic leukemia (HL-60) cells and the Lewis lung carcinoma (LLC) cells from ATCC were purchased from the Chinese Academy of Sciences and were grown in RPMI1640 medium supplemented with 10% (v/v) fetal bovine serum (FBS) in a humidified atmosphere containing 5% CO2 and 95% air at 37 °C. To obtain N1-like neutrophils (HL-60N1), HL-60 cells were seeded at 2 × 10^5^ cells/mL in medium supplemented with 10 μM all-trans retinoic acid (ATRA) for 7 days in a flask [28], changing the medium after 3 days. To obtain N2-like neutrophils (HL-60N2), HL-60N1 cells were stimulated with 100 ng/mL of TGF-β1 for 3 h prior to further assays [19]. HL-60N1 (CD66b^−^) and HL-60N2 (CD66b^+^) cells were magnetically separated by anti-CD66b microbeads (Miltenyi).

HL-60N1 or HL-60N2 cells were seeded in 6-well plate and incubated for 1 h with the indicated concentrations of emodin and then challenged with 50 nM PMA for 3 h. Cell proliferation was examined by MTT according to our previous method [29]. Cells were analyzed by Laser holographic cell imaging and analysis system (HoloMonitor M4, Phiab, Sweden). The phagocytic function of neutrophils was detected by neutral red uptake [30] and *E. coli* phagocytosis [31]. Cell autophagic proteins were tested by western blot. Cell apoptosis was examined by the binding of ANXV-FITC to phosphatidylserine using an automated cell counter and analysis system (Nexcelom Cellometer X2, Nexcelom, USA). ROS was detected by DCFH-DA (2′,7′-dichlorodihydrofluorescein diacetate) using a fluorescence photometer. NET formation was observed by DAPI staining [32].

### Western blot

The intracellular protein was extracted from HL-60N1 (CD66b^−^) and HL-60N2 (CD66b^+^) cells in cell lysis buffer. Equal amounts of protein were separated via 12% sodium dodecyl sulfate–polyacrylamide gel electrophoresis, electroblotted onto nitrocellulose membranes, and probed with antibodies against P62, LC3-B, and β-actin. Antibody binding was detected via enhanced chemiluminescence according to the manufacturer’s instructions (Pierce, Rockford, IL). Band density was quantified using ImageJ software (NIH, Bethesda, MD, USA) and normalized to the corresponding control group.

### Animals

Ten-week-old female ICR mice were obtained from Henan Provincial Medical Laboratory Animal Center. All mice were housed in individually ventilated cages (lights on 7:00 AM to 7:00 PM). Animals were fed standard rodent chow and water. All animal procedures were approved by the Animal Experimentation Ethics Committee of Henan University (permission number HUSAM 2016-288), and all procedures were performed in strict accordance with the Guide for the Care and Use of Laboratory Animals and the Regulation of Animal Protection Committee to minimize suffering and injury.

### Urethane-induced lung carcinogenesis model

Urethane (600 mg/kg body weight), alone or in combination with Ly6G mAb (500 µg/mouse), was injected intraperitoneally (i.p.) into ICR mice (thirty mice per group) once a week for 4, 8 or 10 weeks, according to our previous protocol [33]. Following the first urethane injection, mice received emodin (10 mg/kg/day) via intragastric administration once a day for 4, 8 or 17 weeks. At 5, 9 and 18 weeks after the first urethane injection, the blood from the retro-orbital sinuses was collected in anticoagulant tubes for platelet counts by a blood cell analyzer, and platelet-rich and platelet-free plasma were extracted for measurement of coagulation parameters (PT, APTT, PAgT and FIB) using a coagulation analyzer. The mice were sacrificed under anesthesia with pentobarbital sodium (45 mg/kg), the alveolar fluid was collected by inserting a cannula into the trachea with three sequential injections of 1 mL PBS, supernatant was used for cytokine assay (IFN-γ, IL-12, ROS, TNF-α, IL-6 and TGF-β1) after centrifugation, and the centrifuged cells were resuspended in 0.9% sterile saline for total cell counts and added into a magnetic cell sorting column for neutrophile collection based on anti-LY6G-coated beads (providing > 90% purity, as assessed by Wright-Giemsa stain) Neutrophil immunophenotypes were analyzed by FITC-conjugated anti-mouse CD66b staining using an automated cell counter and analysis system (Nexcelom Cellometer X2, Nexcelom, USA).

To examine the bleeding time (BT), the mice were anesthetized using intraperitoneal injection of pentobarbital sodium (45 mg/kg). A 5-mm tail tip was cut off and the bleeding time was recorded when bleeding had stopped for more than 30 s.

To observe NETs, the neutrophils were incubated in RPMI1640 medium in 96-well plates for 30 min, then stimulated with 100 nM PMA for 4 h, fixed in 4% paraformaldehyde, stained by DAPI and analyzed under a wide-field fluorescent microscope (Olympus BX60) with MetaMorph software.

A part of each lung was preserved in 10% buffered formalin and routinely embedded in paraffin. For lung NETs, lung sections were stained by immunohistochemistry (anti-mouse-Cit-H3, anti-mouse-PAD4) according to our previous method [34]. The total immunohistochemical score was calculated by the intensity score and proportion score by excluding the primary antibody and IgG matched serum, respectively as positive and negative controls.

For H&E staining, each lung section was systematically scanned under a microscope, and five successive fields were graded using a lesion score (LS) based on the area of involved lesions: grade 0, no lesions; grade 1, lesions occupy less than 10% of the scanned field; grade 2, lesions occupy 10–30% of the scanned field; grade 3, lesions occupy 30–50% of the scanned field; grade 4, lesions occupy more than 50% of the scanned field.

IFN-γ, IL-12, ROS, TNF-α, IL-6 and TGF-β1 were determined by ELISA kits, according to the manufacturer’s protocols. The results were calculated from linear curves obtained using the Quantikine kit standards.

### Tumor allograft model

LLC cells were used for tumor allograft experiments. 200 µL saline containing 1 × 10^6^ cells was injected subcutaneously into the lateral axilla of mice to establish tumor allografts. One day after tumor inoculation, in vitro HL-60-drived HL-60N1 or HL-60N2 cells (2 × 10^6^ cells in 200 µL saline), were injected intravenously into mice once a week for 3 weeks; simultaneously, mice received emodin (10 mg/kg once a day via intragastric administration) or DNase I (200 U/mouse once a week via intraperitoneal administration) for 3 weeks (ten mice per group). Tumor size was monitored twice a week with calipers and calculated as the length × width^2^/2. On the 22nd day after tumor inoculation, the mice were euthanized; the blood for blood cell counts measurement of coagulation parameters (PT, APTT, PAgt and FIB). The tumors were removed and weighed. Tumor neutrophils were magnetically separated by anti-LY6G-coated beads, and their expressions of MPO and CD66 were examined by western blot. CD66^+^ cells were also counted by an automated cell counter and analysis system.

### The regulatory mechanism of emodin on neutrophils

The gene expression profiles GSE43254 was obtained from the Gene Expression Omnibus (GEO) database (https://www.ncbi.nlm.nih.gov/geo/query/acc.cgi?acc=GSE43254), up- and down-regulated genes related to tumor-associated neutrophils (N2) were identified using GEO2R, and the human structures of these differential proteins were collected from the protein data bank (PDB) for docking. The chemical structure of emodin was obtained from PubChem and the docking exercise was conducted using the online software systemsDock (http://systemsdock.unit.oist.jp) with the auto-movement of nonspecified protein structures. Docking scores over 6 were regarded as the potential targets for emodin. The gene ontology (GO) and Kyoto Encyclopedia of Genes and Genomes (KEGG) enrichment analyses were performed for the potential targets using the Database for Annotation, Visualization and Integrated Discovery (DAVID) (https://david.ncifcrf.gov) and the online software Omicshare (http://www.omicshare.com). The protein–protein interaction (PPI) among these potential targets was constructed using the STRING database (https://string-db.org/cgi/input.pl) and the hub genes were identified using Cytoscape.

### Statistical analyses

The data was statistically analyzed using GraphPad Prism, Version 5.0 (San Diego, CA, USA) and presented as the mean ± SD. The differences between the two groups were evaluated using a t-test. A P value of less than 0.05 was considered statistically significant.

## Results

### Emodin selectively regulates neutrophils in vitro

**Fig. 1:**
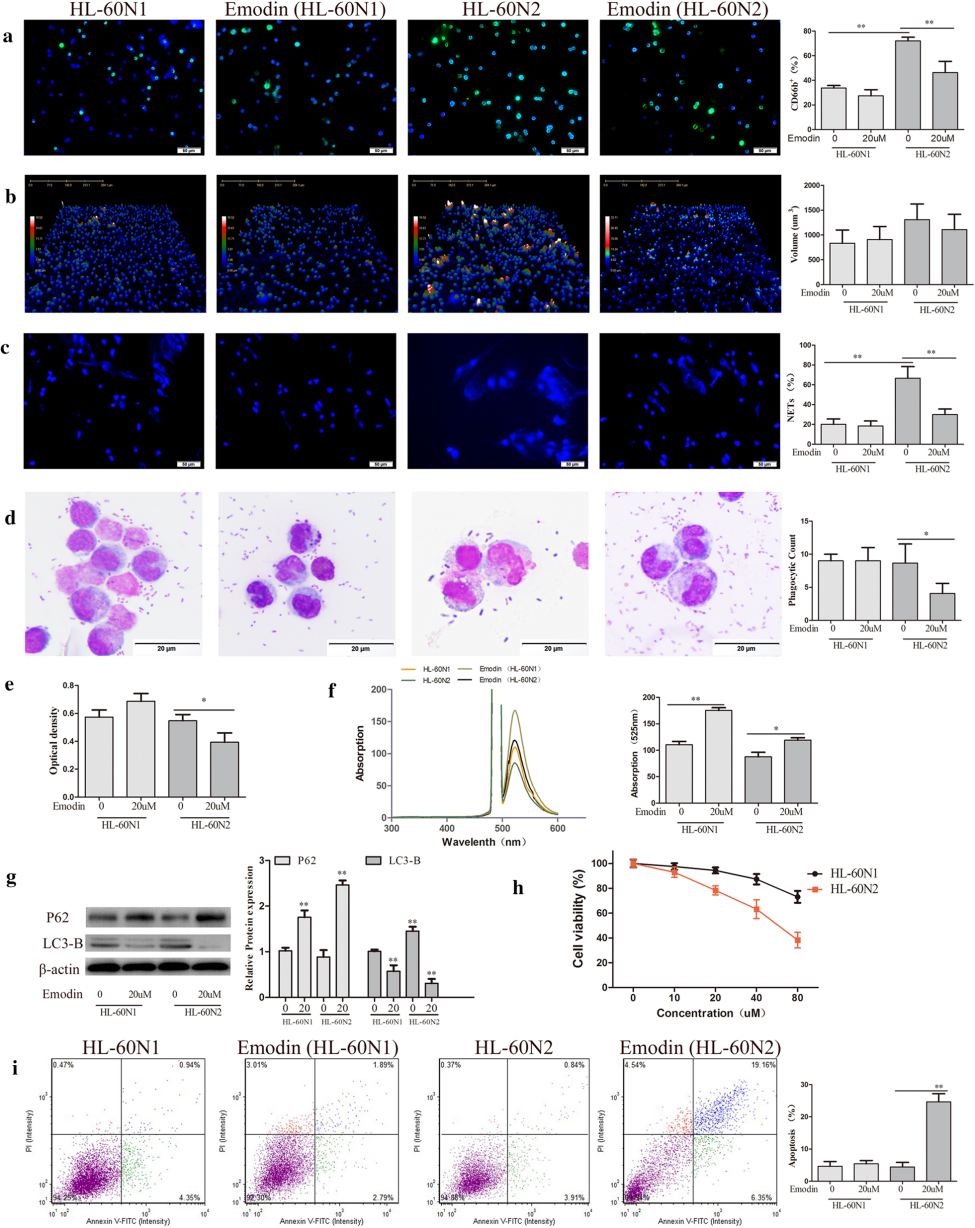
Emodin selectively regulates N2 neutrophils in vitro. **a** CD66b expression in HL-60-differentiated N1 and N2 neutrophils analyzed by FITC-conjugated anti-mouse CD66b staining (n = 5, ×40). **b** Morphological changes N1 and N2 neutrophils analyzed by Laser holographic cell imaging and analysis system (n = 5, ×40). **c** NET formation analyzed by DAPI staining (n = 5, ×40). **d** Neutrophil phagocytosis detected by the Giemsa staining (n = 5, ×100). **e** Neutral red uptake examined with Microplate reader (n = 5). **f** ROS detected by DCFH-DA (n = 5). **g** Cell autophagy-associated proteins examined by Western blot (n = 3). **h** Cell viability examined by MTT (n = 5). **i** Cell apoptosis detected by Annexin V-FITC apoptosis kit (n = 5). The data present mean ± SD, the experiments were repeated 3 times, and statistical significance was determined by a t-test. *P < 0.05, **P < 0.01

HL-60 cells have high plasticity and are widely used in the study of phenotype and function of neutrophils [35]. To explore the effect of emodin on neutrophils, we induced the differentiation of HL-60 cells into N1-like neutrophils (HL-60N1) and N2-like neutrophils (HL-60N2). Compared to HL-60N1, HL-60N2 cells had high levels of surface CD66b expression (Fig. 1a) with the morphological changes (Fig. 1b) and were easy to form NETs (Fig. 1c). There was no difference in E.coli phagocytosis (Fig. 1d) and neutral red uptake (Fig. 1e) between HL-60N1 and HL-60N2, however, HL-60N2 had a reduction in ROS (Fig. 1f) and an increase in autophagy (Fig. 1g). When administered at the dose (20 μM) that has no effect on cell viability in HL-60N1 (Fig. 1h), emodin increased ROS and decreased autophagy (Fig. 1f, g) and thus induced apoptosis (Fig. 1i) in HL-60N2 with the morphological changes (Fig. 1b). In addition, emodin treatment also decreased NETs in HL-60N2 cells (Fig. 1c). The experiments were repeated 3 times and the similar results were obtained.

### Urethane-induced lung carcinogenesis was positively correlated with the neutrophil-associated coagulation

**Fig. 2:**
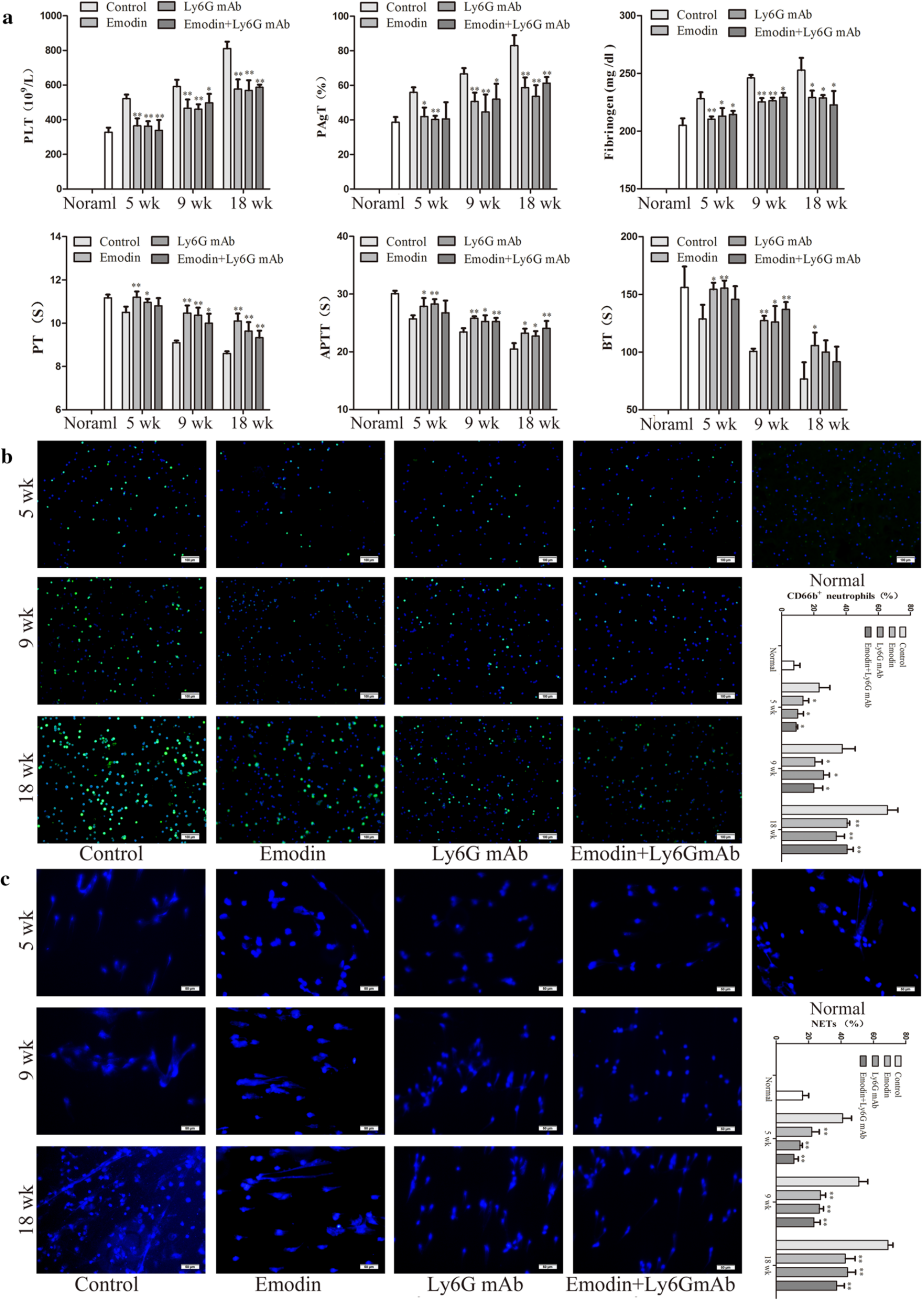
Emodin suppresses N2 neutrophiles to attenuate hypercoagulation in a time-dependent manner during urethane-induced lung carcinogenesis. **a** Hypercoagulation indicated by platelet counts, platelet aggregation, fibrinogen (FIB), prothrombin time (PT), activated partial thromboplastin time (APTT) and bleeding time (BT) (n = 6). **b** CD66b expression in alveolar neutrophils (n = 5, ×20). **c** NET formation in alveolar neutrophils analyzed by DAPI staining (n = 5, ×40). The data present Mean ± SD, the experiments were repeated 3 times, and statistical significance was determined by a t-test. *P < 0.05, **P < 0.01 vs control

**Fig. 3:**
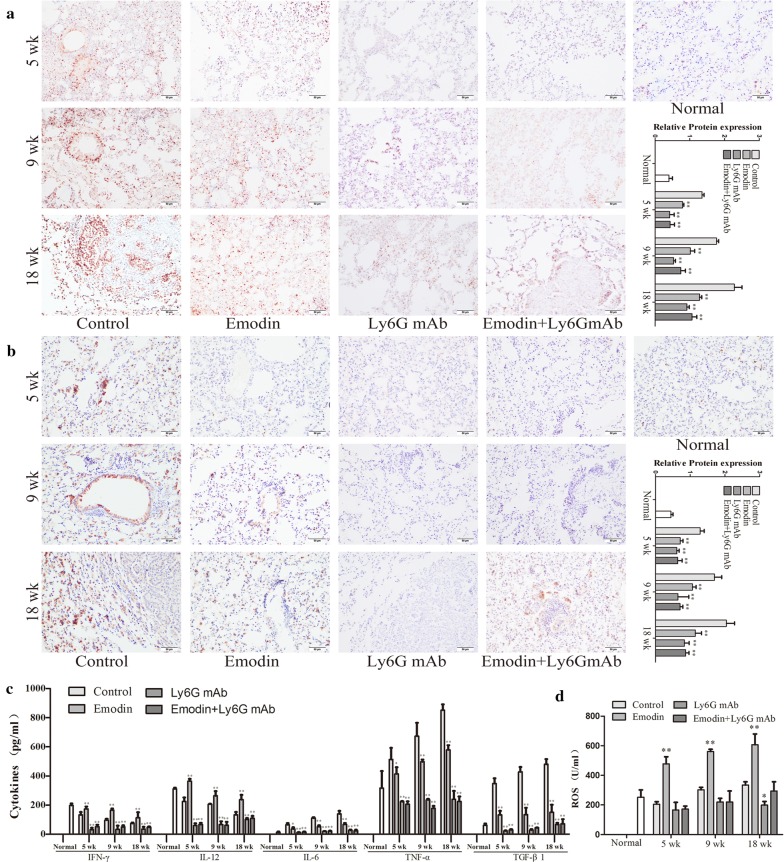
Emodin attenuates NETs and changes cytokines and ROS during urethane-induced lung carcinogenesis. **a** NETs in lung sections indicated by Cit-H3 examined by immunohistochemistry (n = 5, 40 ×). **b** NETs in lung sections indicated by PAD4 examined by immunohistochemistry (n = 5, 40 ×). **c** Cytokine levels in alveolar cavity examined by ELISA (n = 6). **d** ROS in alveolar cavity examined by ELISA (n = 6). The data present Mean ± SD, the experiments were repeated 3 times, and statistical significance was determined by a t-test. *P < 0.05, **P < 0.01 *vs* control

**Fig. 4:**
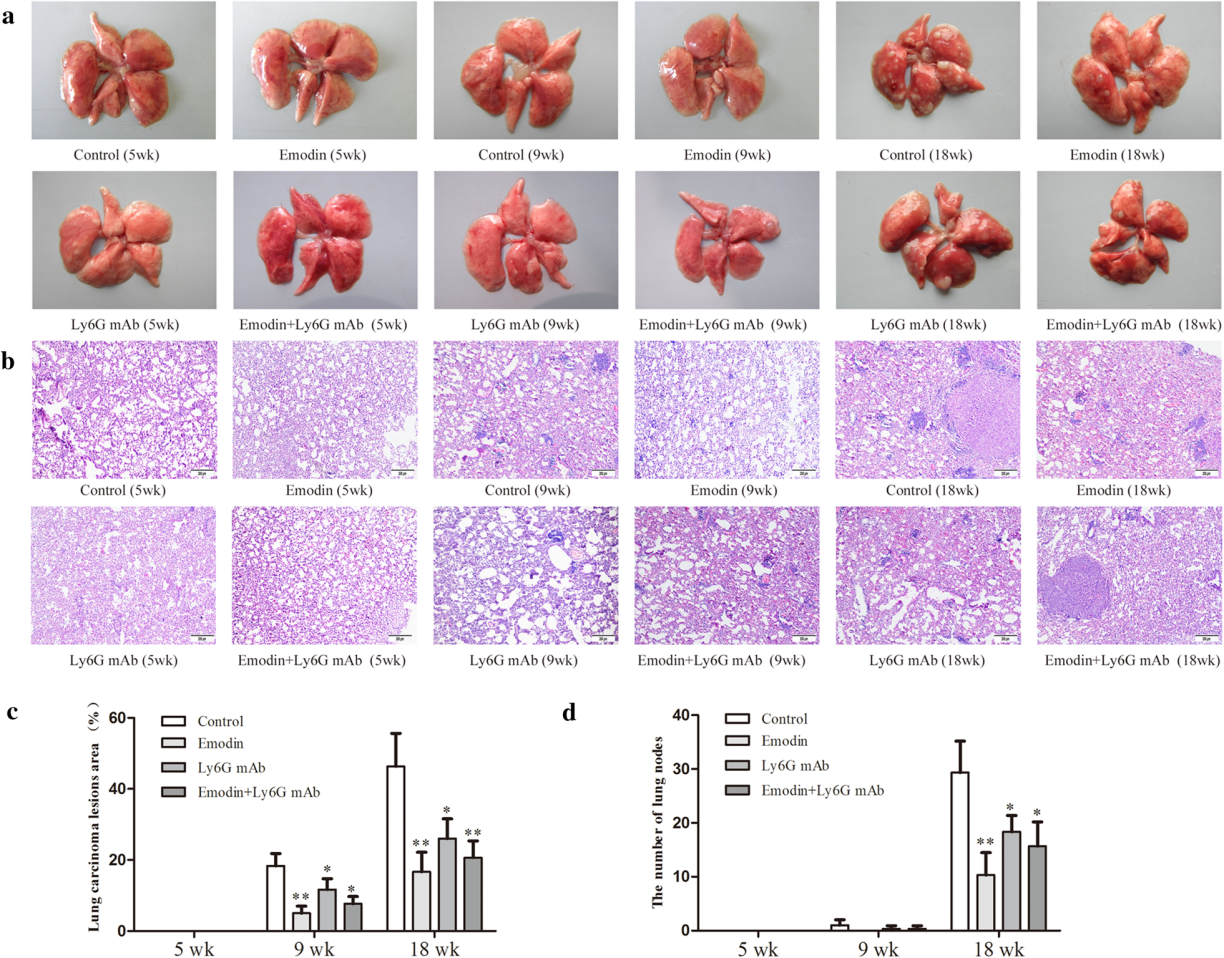
Emodin prevents lung carcinoma lesions and carcinogenesis during urethane-induced lung carcinogenesis. **a** The whole lung in naked eye (n = 10). **b** HE staining of lung tissues (n = 10, ×10). **c** The area of involved lesions in HE staining (n = 10). **d** The number of nodes in lung surface (n = 10). The data present mean ± SD, the experiments were repeated 3 times, and statistical significance was determined by a t-test. *P < 0.05, **P < 0.01 *vs* control

Urethane-induced mouse lung cancer model is used for studying basic lung tumor biology and finding new tumor intervention strategies. In this study, urethane at the dose of 600 mg/kg body weight was injected intraperitoneally (i.p.) once a week. At 5 weeks, prior to lung carcinogenesis, the four urethane injections led to obvious hypercoagulation in control group, presenting as an increase in platelet counts, platelet aggregation, fibrinogen (FIB) production, and a reduction in prothrombin time (PT), activated partial thromboplastin time (APTT) and bleeding time (BT) (Fig. 2a) which were positively correlated with N2 neutrophils (CD66b^+^ cells) and NETs (Fig. 2b, c). Immunohistochemistry confirmed NET formation in lung tissues, as indicated by staining for histone Cit-H3 (Fig. 3a) and peptidyl-arginine deiminase 4 (PAD4) (Fig. 3b). At 9 weeks, the eight urethane injections resulted in visible lung carcinoma lesions (Fig. 4b) under a microscope accompanied by the aggravated hypercoagulation, N2 neutrophils and NETs (Fig. 2), but no lung cancer nodes were visible to the naked eye (Fig. 4a). At 18 weeks, ten urethane injections led to the development of lung cancer nodes visible to the naked eye (Fig. 4a) accompanied by further aggravated hypercoagulation, N2 neutrophils and NETs (Fig. 2). The number of lung cancer nodes was 28.2 ± 5.2 (Fig. 4d), regardless of the heterogeneity of tumor histology in the experimental group. ELISA showed that the levels of IFN-γ, IL-12 and ROS decreased and the levels of TNF-α, IL-6 and TGF-β1 increased in alveolar cavities in control mice compared to normal mice (Fig. 3c, d). As expected, neutrophil depletion using the Ly6G mAb following urethane injection significantly inhibited lung carcinoma lesions and carcinogenesis accompanied by the decreased hypercoagulation, N2 neutrophils and NETs (Figs. 2, 3, 4). The experiments were repeated 3 times and the similar results were obtained.

### Emodin regulates neutrophils to prevent hypercoagulation and urethane-induced lung carcinogenesis

To prove the preventive effects of emodin on hypercoagulation and lung carcinogenesis, the mice received emodin after the first urethane injection. At five weeks, compared to the control group, emodin treatment resulted in a reduction in coagulation accompanied by the decreased N2 neutrophils and NETs (Fig. 2a–c). The NET preventive efficacy of emodin was further verified in immunohistochemical staining of histone Cit-H3 and peptidyl-arginine deiminase 4 (PAD4) (Fig. 3a, b). At 9 and 18 weeks, respectively, there were slighter carcinoma lesions and less lung cancer nodes in emodin group than those in control group (Fig. 4), which were in line with the decreased hypercoagulation, N2 neutrophils and NETs (Fig. 2) and the improved cytokine levels indicated by the increased IFN-γ, IL-12 and ROS and the decreased TNF-α, IL-6 and TGF-β1 (Fig. 3c, d). Neutrophil depletion using the Ly6G mAb significantly attenuated the preventive effects of emodin on hypercoagulation and lung carcinogenesis (Figs. 2, 3, 4). The experiments were repeated 3 times and the similar results were obtained.

### Emodin exhibits neutrophil-regulating capacity in a Lewis lung cancer allograft model

**Fig. 5:**
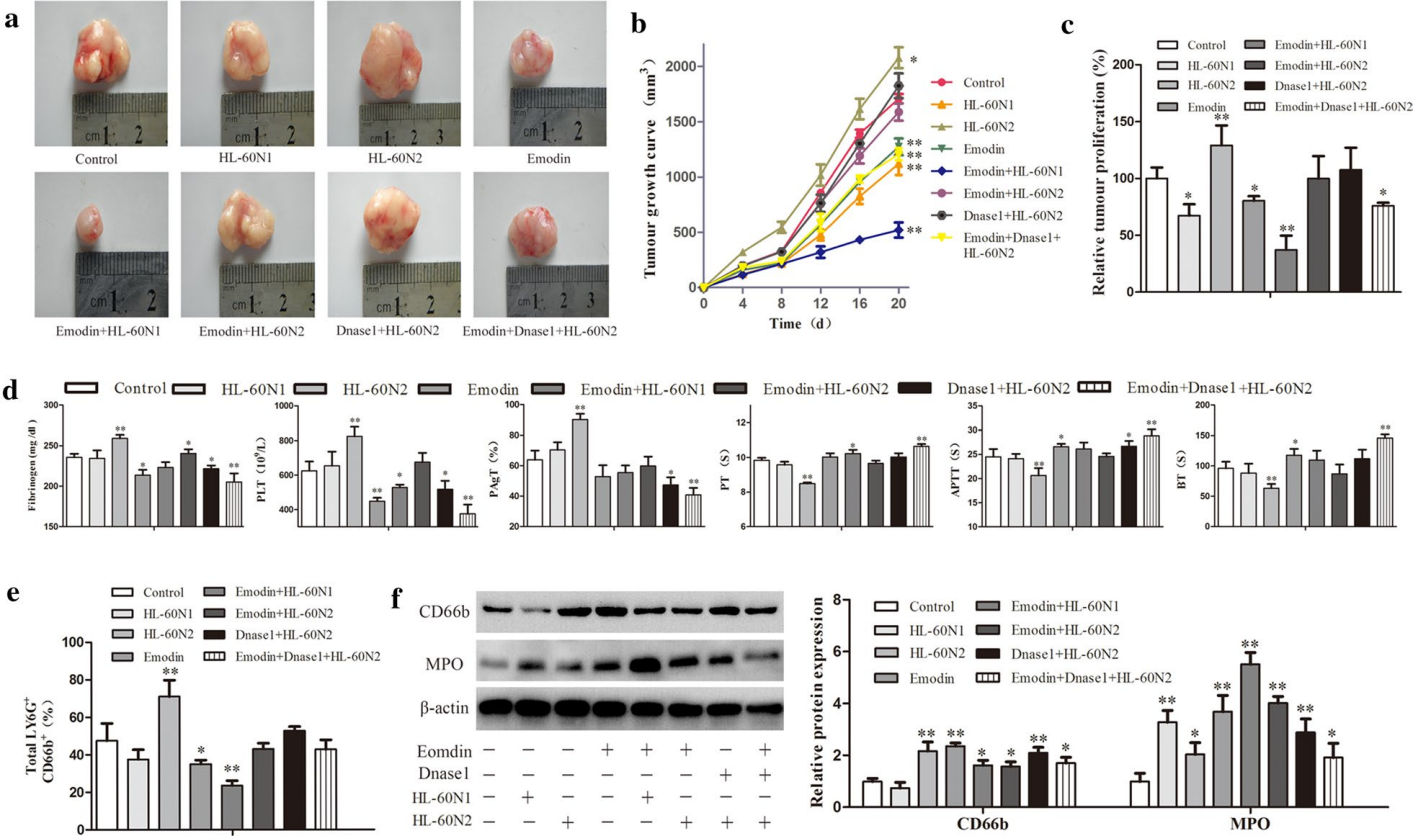
Emodin exhibits neutrophil-regulating capacity in a Lewis lung cancer allograft model. **a** Tumor appearance. **b** The curve of tumor growth (n = 10). **c** The relative tumor proliferation (n = 10). **d** Hypercoagulation indicated by platelet counts, platelet aggregation, fibrinogen (FIB), prothrombin time (PT), activated partial thromboplastin time (APTT) and bleeding time(BT)(n = 6). **e** CD66b+ cells in intratumor neutrophils analyzed using fluorescent cell count (n = 5). **f** The protein expression in intratumor neutrophils examined by western blot (n = 3). The data present Mean ± SD, the experiments were repeated 3 times, and statistical significance was determined by a t-test. *P < 0.05, **P < 0.01 vs control

To further prove the relativity of emodin-ameliorated hypercoagulation and lung carcinogenesis to N2 neutrophils, we established an allograft model of Lewis lung cancer and used emodin alone or in combination with HL-60N1 or HL-60N2 cells to treat the model mice. The transfer of HL-60N2 cells promoted tumor growth, whereas the transfer of HL-60N1 cells or the degradation of NETs with DNAse I prevented tumor growth (Fig. 5a–c). Emodin alone suppressed tumor growth by 20%, but synergistically prevented tumor growth in combination with HL-60N1 cells or DNAse I and attenuated the tumor-promoting efficacy of HL-60N2 cells (Fig. 5a–c), which was consistent with hypercoagulation that was promoted by HL-60N2 cells and attenuated by emodin and DNAse I (Fig. 5d). CD66b^+^ cell count (Fig. 5e) and western blot (Fig. 5f) further confirmed the N2 neutrophil-dependent effect of emodin on hypercoagulation and lung carcinogenesis. The experiments were repeated 3 times and the similar results were obtained.

### Emodin regulates neutrophils by targeting multi-protein network

**Fig. 6:**
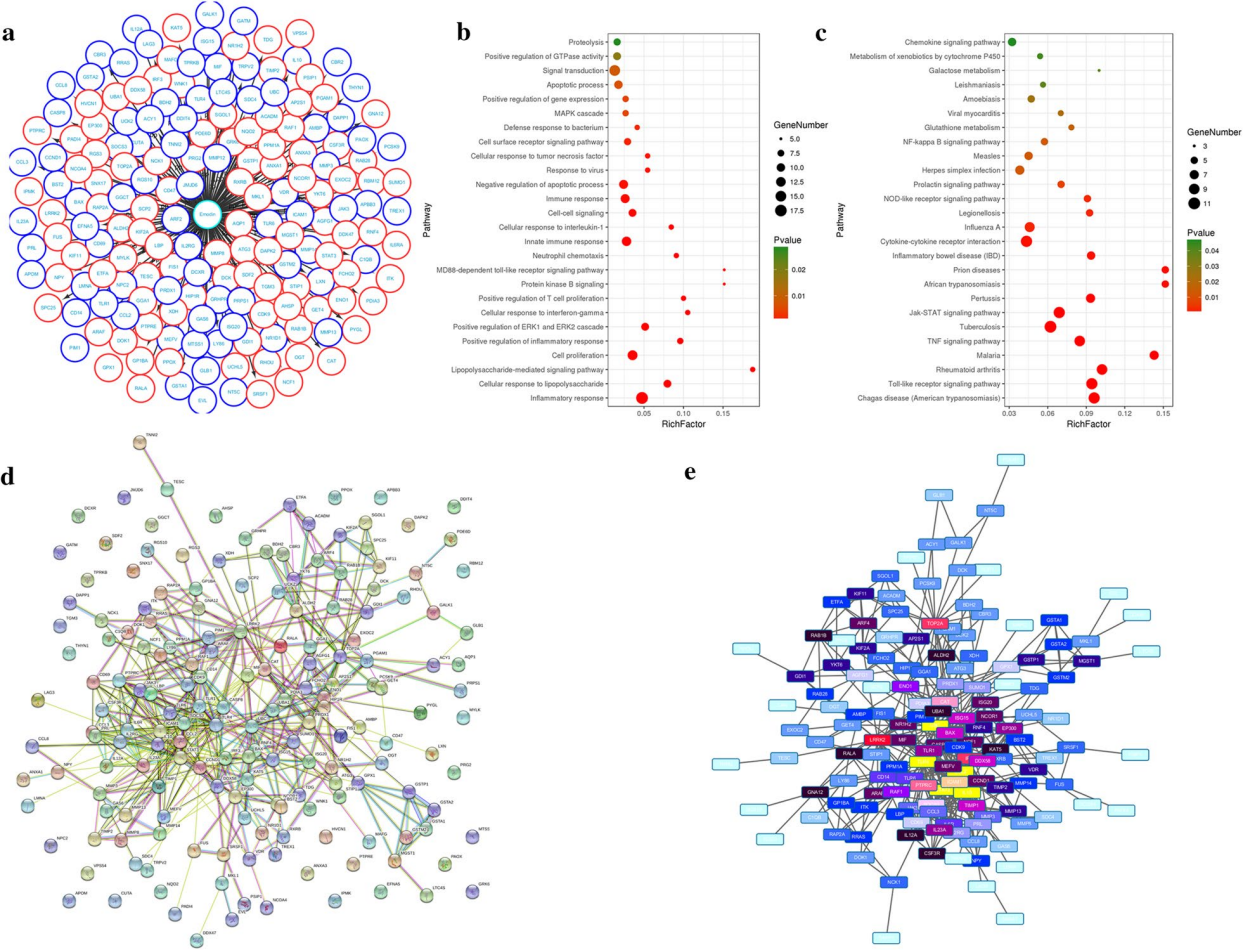
Emodin regulates N2 neutrophils by targeting multi-protein network. **a** The potential targets of emodin with docking score > 6.0. **b** GO enrichment analysis performed by DAVID and visualized by Omicshare. **c** KEGG enrichment analysis performed by DAVID and visualized by Omicshare. **d** The PPI network constructed by the STRING database. **e** The hub genes identified by Cytoscape

We queried 853 up-regulated genes (LogFC ≥ 1.5, *P *< 0.05) and 1023 down-regulated genes (LogFC ≤ − 1.5, *P *< 0.05) related to N2 neutrophils and obtained 237 targeting information (Fig. 6a). 177 of the potential targets with docking score > 6.0 (pKd/pKi) were selected for GO and KEGG analyses (Table 1). The hypergeometric distribution count > 2 and *P* < 0.05 were set as threshold criteria to identify the functional gene ontology and pathway. GO enrichment analysis indicated that the potential targets of emodin were primarily associated with the “inflammatory response”, “signal transduction”, “cell proliferation”, “innate immune response” and “negative regulation of apoptotic process” terms (Fig. 6b). KEGG enrichment analysis revealed that the potential targets of emodin were significantly enriched in the “Toll-like receptor signaling pathway”, “Jak-STAT signaling pathway” and “Cytokine-cytokine receptor interaction” terms (Fig. 6c). The PPI network (Fig. 6d) identified 4 key genes (IL-10, TLR-4, START3 and CCL2), which were hub genes for emodin (Fig. 6e).

**Table 1:** 177 of the potential targets with docking score > 6.0

Protein name	PDB ID	Docking scores (pKd/pKi)	Protein name	PDB ID	Docking scores (pKd/pKi)	Protein name	PDB ID	Docking scores (pKd/pKi)
Up-regulated genes								
CCL3	3FPU	7.775	IL23A	3D85	6.835	MMP3	1HY7	6.668
IL10	2ILK	7.354	NT5C	2I7D	6.826	ISG15	1Z2M	6.664
BDH2	2AG5	7.183	CCL8	1ESR	6.824	TPRKB	3ENP	6.659
GGCT	3CRY	7.093	EFNA5	1SHX	6.824	MMP12	1Y93	6.512
GSTM2	2C4J	7.062	PRPS1	2H06	6.824	PAOX	1W6G	6.509
DCXR	3D3W	6.981	MTSS1	2D1L	6.822	ACY1	1Q7L	6.431
CBR2	1CYD	6.941	GAS6	1H30	6.81	JAK3	3LXL	6.417
ICAM1	1IAM	6.941	CCL2	1DOK	6.8	JMJD6	3K2O	6.414
AMBP	1BIK	6.929	PRDX1	3HY2	6.799	LY86	3M7O	6.389
SDC4	1OBY	6.929	DDIT4	3LQ9	6.79	TLR1	2Z7X	6.375
C1QB	2WNV	6.922	LMNA	1IFR	6.775	CUTA	1KR4	6.354
CD69	3HUP	6.92	RRAS	2FN4	6.775	PIM1	1XWS	6.342
TRPV2	2ETB	6.915	BST2	3NWH	6.761	EVL	1QC6	6.34
NPC2	1NEP	6.907	CD14	1WWL	6.751	TLR6	3A79	6.31
UCK2	1UJ2	6.903	DAPP1	1FAO	6.747	GATM	1JDW	6.302
GSTA2	1ML6	6.9	IL12A	1F45	6.746	PCSK9	2QTW	6.219
LAG3	2FO1	6.89	SOCS3	2HMH	6.742	APOM	2WEW	6.213
BAX	2G5B	6.888	TREX1	2IOC	6.731	GLB1	3G46	6.207
GSTA1	1K3Y	6.881	ARF2	1J2 J	6.73	ISG20	1WLJ	6.203
TLR4	2Z62	6.871	MEFV	2WL1	6.709	RGS10	2IHB	6.192
NR1D1	1A6Y	6.87	THYN1	3EOP	6.685	VDR	1IE9	6.159
CBR3	2HRB	6.869	LTC4S	2UUI	6.681	CCND1	2W96	6.144
APBB3	2DYQ	6.864	CASP8	1QTN	6.678	MMP14	3C7X	6.135
LXN	2BO9	6.855	MMP13	830C	6.677	TNNI2	1A2X	6.118
PRL	3D48	6.855	IL2RG	2B5I	6.676			
GRHPR	2GCG	6.841	MIF	3DJH	6.673			
Down-regulated genes								
STAT3	1BG1	7.945	RNF4	2XEU	6.841	KIF11	1X88	6.645
NCOR1	3KMZ	7.085	AGFG1	2OLM	6.837	DCK	1P5Z	6.641
NCOA4	1T5Z	7.084	FUS	1SVF	6.837	AHSP	1Z8U	6.629
ACADM	1EGD	7.073	ANXA3	1AXN	6.836	NR1H2	1PQ9	6.624
KAT5	2OU2	7.065	RBM12	2EK1	6.834	FCHO2	2V0O	6.593
SDF2	3MAL	7.065	IPMK	2IEW	6.832	NQO2	2QWX	6.592
TDG	1WYW	7.021	SCP2	2SAS	6.824	RGS3	3FBK	6.584
MYLK	2O5G	7.005	OGT	1MGT	6.82	AQP1	1J4N	6.582
TOP2A	1ZXM	7.002	HVCN1	3A2A	6.816	MMP8	1I76	6.484
ITK	3MIY	6.98	PDE6D	1KSH	6.81	MKL1	2V52	6.459
UBA1	3CMM	6.971	LRRK2	2ZEJ	6.809	PTPRE	2JJD	6.438
DDX58	3LRR	6.966	CDK9	3MI9	6.808	GET4	2WPV	6.414
NCF1	1KQ6	6.944	GGA1	1J2J	6.8	XDH	3NRZ	6.407
PYGL	3DDS	6.936	IL6RA	1N26	6.797	TESC	1NJK	6.398
RAP2A	1KAO	6.935	MGST1	2H8A	6.786	GNA12	1ZCA	6.384
GRK6	2ACX	6.924	PPOX	2IVD	6.784	YKT6	3KYQ	6.377
CD47	2JJS	6.923	SRSF1	3BEG	6.773	ARAF	8ABP	6.346
KIF2A	2GRY	6.922	SNX17	3LUI	6.772	SGOL1	3FGA	6.336
RAB28	2HXS	6.919	CSF3R	1CD9	6.759	PPM1A	1A6Q	6.331
IRF3	3A77	6.916	MAFG	3A5T	6.756	HIP1R	1R0D	6.319
SUMO1	2UYZ	6.912	RALA	1U8Z	6.756	LBP	2HPS	6.317
GP1BA	1P9A	6.903	NCK1	2CI9	6.742	RXRB	1H9U	6.283
PDIA3	2H8L	6.896	PADI4	2DEW	6.738	GDI1	2BCG	6.27
VPS54	3N1E	6.887	STIP1	1ELW	6.738	PTPRC	1YGR	6.268
ATG3	2DYT	6.883	EXOC2	1UAD	6.719	ENO1	2AL1	6.225
NPY	1R9N	6.881	TIMP2	2E2D	6.719	WNK1	3FPQ	6.169
SPC25	2FTX	6.878	EP300	3BIY	6.707	CAT	1Q23	6.105
RAF1	1C1Y	6.87	PRG2	1H8U	6.701	PGAM1	1YFK	6.07
AP2S1	2VGL	6.859	TGM3	1VJJ	6.701	DDX47	3BER	6.062
FIS1	2PQR	6.855	ETFA	1O97	6.694	PSIP1	2B4J	6.019
GPX1	2V1M	6.854	DOK1	2V76	6.679	ANXA1	1HM6	6.014
DAPK2	2A2A	6.853	UCHL5	3A7S	6.665	GSTP1	2A2R	6.007
RHOU	2Q3H	6.853	ALDH2	1O04	6.655	RAB1B	3NKV	6.007

## Discussion

Neutrophils are categorized into two subsets, high-density neutrophils (HDNs) and low-density neutrophils (LDNs), characterized by distinct morphology, phenotype and function [36]. HDNs are a homogenous population of mature neutrophils and can acquire a cytotoxic phenotype to exert anti-tumorigenic (N1) function in response to a variety of stimuli [37]. In contrast, LDNs have a larger volume than HDNs, and comprise a heterogeneous population of immature and mature neutrophils and can display pro-inflammatory and immunosuppressive properties to exert pro-tumorigenic (N2) function [38]. One way to classify HDNs and LDNs is by their sedimentation properties in density gradients [39]. In fact, both of HDNs and LDNs contain diverse cell populations with different functions. In this study, we use N2 cell surface marker-CD66b and NET formation to classify HDNs and LDNs based on the literature [25, 40]. We found that urethane induced obvious hypercoagulation which was positively correlated with lung N2 neutrophils and NETs before lung carcinogenesis, and that there were the aggravated hypercoagulation and lung N2 neutrophils as well as NETs after lung carcinoma lesions, whereas neutrophil depletion resulted in the decreased hypercoagulation and lung carcinogenesis, indicating a loop of N2 activation-NETs-hypercoagulation-carcinogenesis during urethane induces lung cancer. We suppose that neutrophil phenotypes are dynamic and can be modulated by small molecular compounds, our results showed that emodin treatment significantly attenuated lung N2 neutrophils and NETs inconsistent with the decreased hypercoagulation and carcinogenesis, suggesting a relativity of emodin-ameliorated hypercoagulation and lung carcinogenesis to N2 neutrophils.

Cancer patients often exhibit hypercoagulation, which is one of the most common causes of cancer-related death [41–43]. A recent study suggested that neutrophils were essential for the initiation and propagation of cancer-associated thrombosis by releasing NETs, indicating a novel link between neutrophils and carcinogenesis, as well as cancer-associated hypercoagulation [44, 45]. Neutrophils are the first immune cell type to be recruited to the inflammation site and exert the first line defense against a variety of pathogens [18]. Abundant evidence suggest that N2 neutrophils are an immunosuppressive subset of neutrophils that acts as a key regulator at the various stages of tumor development [39]. Considering the anti-tumorigenic function of neutrophils (N1), we suppose that regulating of N2 to N1, rather than complete depletion of neutrophils, is a better approach for tumor prevention. In this study, we found that emodin at the optimal dose selectively suppressed N2 neutrophils in vitro. The relativity of emodin-ameliorated hypercoagulation and lung carcinogenesis to N2 neutrophils was also further proved in Lewis lung cancer allograft model, in which emodin did not attenuate significantly tumor growth but prevented synergistically hypercoagulation and tumor development in combination with HL-60N1 cells or DNase I, which tested our hypothesis and also suggested the dual roles of hypercoagulation as an initiator and a promoter in carcinogenesis. Although emodin has been extensively investigated for cancer prevention and treatment, there is insufficient information related to the mechanism by which emodin exerts its anticancer properties in different cancers [46]. It has been proved that emodin can modulate mitochondrial apoptosis pathway, mitotic catastrophe and P2Y receptors in different cancers [47]. Xing et al. revealed that there were significant changes in many endogenous metabolites after emodin exposure concerning oxidative stress and disturbances in amino acid and energy metabolism [48]. In this study, we try hard to explain the mechanism by which emodin regulates N2 neutrophils to attenuate hypercoagulation and lung carcinogenesis. Network pharmacology indicated that the potential targets of emodin regulating N2 neutrophils were primarily associated with the “inflammatory response”, “signal transduction”, “cell proliferation”, “innate immune response” and “negative regulation of apoptotic process, indicating a function of emodin targeting multi-protein network, such as “Toll-like receptor signaling pathway”, “Jak-STAT signaling pathway” and “Cytokine-cytokine receptor interaction”, whereby it regulates neutrophil phenotypes to maintain system homeostasis for cancer prevention. Further mechanism studies are necessary to verify its medicinal applications.

Cancer chemoprevention is an important prophylactic strategy for reduction of cancer-related death [49]. Despite substantial progress and successful precedent, such as the use of HPV vaccine for prevention of cervical cancer, cancer chemoprevention has faced many challenges and needs to be used more broadly [50, 51]. One misunderstanding is that the molecular targets of chemopreventive agents must coincide with the targets of anti-cancer therapies [52]. It is very important for medical professionals to fully appreciate the benefits of cancer chemoprevention and the need for the development of precise strategies, as well as for understanding the mechanistic aspects of these strategies, in order to prevent progression of precancer lesions into advanced stage cancer [53]. Therefore, the focus of this study was not on tumor killing efficacy of emodin but on effect of emodin on neutrophil phenotypes. Here we propose a specific mechanism by which emodin can delay or reverse the procession of early precancer lesions. Although lacking a precise explanation of mechanism by which emodin regulates N2 neutrophils, our research provides a causative role for coagulation in lung carcinogenesis. With cancer being a global issue and understanding the need for activation of patient’s innate antineoplastic agents, emodin may provide an efficient opportunity as a chemoprevention agent targeting multi-protein network.

## Conclusions

In summary, N2 neutrophils play an important role in hypercoagulation and cancer progression, our findings suggest several potential clinical implications. First, hypercoagulation is the result and cause of carcinogenesis. Second, emodin can selectively suppress N2 to maintain N1 neutrophils for hypercoagulation and cancer prevention. Third, the combination of emodin and N1 neutrophils synergistically prevent hypercoagulation and carcinogenesis and can be a novel strategy for preventing cancer and cancer-associated thrombosis.

## Abbreviations

NET: neutrophil extracellular traps
PMA: phorbol 12-myristate 13-acetate
DAPI: 4′,6-diamidino-2-phenylindole
DCFH-DA: 2′,7′-dichlorodihydrofluorescein
PLT: platelet
PAgT: platelet aggregation test
FIB: fibrinogen
PT: prothrombin time
APTT: activated partial thromboplastin time
BT: bleeding time
HDN: high-density neutrophil
LDN: low-density neutrophil
